# Well-Being and Mental Health in Teachers: The Life Impact of COVID-19

**DOI:** 10.3390/ijerph192215371

**Published:** 2022-11-21

**Authors:** Jerome Flores, Alejandra Caqueo-Urízar, Michael Escobar, Matías Irarrázaval

**Affiliations:** 1 Escuela de Psicología y Filosofía, Universidad de Tarapacá, Arica 1010069, Chile; 2 Centro de Justicia Educacional, CJE, Arica 1010069, Chile; 3 Instituto de Alta Investigación, Universidad de Tarapacá, Arica 1001236, Chile; 4 Centro de Investigación e Intervención Psicosocial, CEINPSI, Universidad de Tarapacá, Arica 1010069, Chile; 5 Millennium Institute for Research in Depression and Personality, MIDAP, Santiago 8380453, Chile

**Keywords:** well-being, teachers, spirituality, depression, anxiety, stress

## Abstract

The impact of the pandemic on teachers’ mental health has also been an important issue. The aim of the study was to analyze the vital impact of COVID-19, spirituality, and the use of social-emotional strategies on teacher well-being, mediated by mental health. The sample was non-random, inviting all teachers in a city North of Chile to participate in the study. The sample consisted of 624 teachers. A total of 74.4% were women and 25.6% were men. The mean age was 44.1 and the standard deviation was 11.9. A total of 56.4% belonged to public schools and 43.6% belonged to subsidized schools. Structural equations were used to analyze the data, finding a mental health mediating effect between the death of a close person, affected areas and family history with life satisfaction. Spirituality and the use of socio-emotional strategies self-applied by the teachers had no direct relationship with their mental health, so their mediating effect in relation to life satisfaction was discarded. Teachers who used social-emotional strategies, as well as those who reported higher levels of spirituality, obtained greater satisfaction with life, both general and specifically. Women had higher levels of depression, anxiety and stress symptomatology, but also higher levels of life satisfaction. The implications are discussed.

## 1. Introduction

The impact of COVID-19 on teachers’ mental health and well-being has received increasing attention. Although crises experienced by the teaching profession are commonplace, the COVID-19 pandemic has undoubtedly had a notable impact inside and outside the classroom on the lives of teachers [1,2,3]. There has been variability in the responses implemented by different countries, both in the extent and/or duration of school lockdowns and closures, as well as vaccination rates [4].

At the onset of the lockdown, almost surprisingly teachers were confronted with the need to manage mostly new technologies to adapt to the online format, as well as demands for emotional containment of their students [5,6,7]. The latter has had a direct effect on the mental health of all members of the educational communities, in addition to the pressure experienced by teachers to recover the learning that has inevitably been delayed as a result of the lack of presence [8].

Evidence of the impact of the pandemic on the mental health of children, adolescents, and their families suggests that teachers are additionally pressured to respond to the need for emotional support from students and their families [8,9].

Longitudinal research found that as the pandemic progressed in 2020, teachers’ mental health was progressively affected. Additionally, teachers belonging to COVID-19 risk groups experienced the greatest deterioration in their mental health [10]. Evidence shows that how teachers feel on a daily basis and their satisfaction can profoundly affect their classroom practices and, among other things, be decisive in both the learning and well-being of their students [11,12]. Meanwhile, poorer teacher mental health and well-being is associated with the risks of experiencing a burnout, which has negative consequences on student achievement as well as student motivation [13] Consequently, teachers’ mental health and well-being should not be seen as an individual concern, but as an organizational one, given their key social role [11]. In addition, no reform or restructuring of the educational system can succeed without the commitment and active contribution of teachers, which is one more reason to safeguard their well-being [14].

It is known from the history of previous pandemics that they affect people’s mental health beyond the end of the pandemic [15]. Therefore, strategies are needed to address their effects on the general population as well as vulnerable populations. Educational communities are at the center of the tension to recover normal functioning.

The aim of the present study is to analyze the life impact of COVID-19, spirituality and the use of social-emotional self-care strategies on teacher well-being, mediated by mental health.

### Literature Review

A review by Agyapong et al. [16] indeed found that burnout, depression, anxiety and stress in teachers are relevant in relation to their well-being and mental health. Even before the pandemic, evidence has been found that teachers’ stress levels may be higher than those of other professions. At the same time, however, job satisfaction may be more rewarding [17]. Strengthening teachers’ internal resources has obtained evidence of being a favorable strategy to promote their well-being, particularly their emotional resources and the meaning of their work [18,19]. On the other hand, the use of self-compassion also seems to play a key role in the care of teachers’ well-being and mental health [20]. Teachers’ use of self-compassion has received increasing evidence [21]. Meanwhile, mindfulness meditation has accumulated considerable previous evidence in reducing teachers’ stress [22].

The pandemic is known to have generated fear among teachers as well, finding that those who expressed the most fear were less willing to reopen schools. Although others have even criticized the need to close schools due to their doubts about the effectiveness of this measure on the decrease in contagion versus the harmful effects on school communities, especially on students’ development [23].

In Chile, a study on the general population during the beginning of the pandemic concluded that the psychological impact of COVID-19 was significant, especially in women and young people [24]. In this larger scenario, it is important to analyze how COVID-19 specifically affects the school teaching population. This study is framed at a time when quarantines ceased to be so strict and gradual presence began to be allowed and promoted in educational institutions.

Usually, the proportion of female teachers is higher than that of male teachers, the former being between 70 and 80% [5]. This reality is also reflected in Chile [25]. Throughout the pandemic, women are the ones who have been most affected, since they are usually the ones who assume a more active role in the daily management of the household. Therefore, even when quarantines end, the exhaustion of going through these experiences may remain, manifesting in symptoms of depression, anxiety or stress, among others [5].

Another study, also in Chile, specifically addressed teachers’ psychological distress as an indicator of well-being in relation to demographic factors, demands and environmental resources [26]. The data from that study were collected during 2020 at the height of the school closure boom, and their results showed that women, as well as teachers who were caring for minors and adults, experienced greater psychological distress. Recently, Lizana and Lera [27] also found high levels of depression, anxiety and stress in Chilean teachers, with higher levels in those under 35 years of age, in women and in public schools.

Kidger et al. [28] found a negative correlation between depression and well-being in teachers. Other researchers have considered psychological well-being directly as their levels of stress, depression and emotional exhaustion [29,30]. One specific aspect of well-being, happiness at work, has been found to predict better mental health in teachers [31].

Among the aspects associated with mental health, it has been considered that spirituality may have a relevant role as a protective factor. Spiritual activities are one of the forms of self-care that teachers have reported to cope with the pandemic [32]. Spirituality is a broad concept, which usually includes belief in a higher power and can occur within or outside religion, which can be understood as a particular type of spirituality [33]. Religiosity has been found to be associated with better mental health and lower burnout in teachers, whether considering religious belief or frequent prayer [33,34]. Spirituality has been found to be associated with better stress management in teachers and lower attrition [35,36].

How the pandemic may impact mental health and teacher well-being should undoubtedly be kept in mind. Fear of COVID-19 contagion has been found to be associated with higher levels of stress and anxiety [2,10]. In the present study, we sought to consider this impact more broadly, considering three aspects: having a history of close people infected with COVID-19, the death of a family member or friend, and the perceived impact on different areas of life.

The use of strategies to cope with emotional stress has been proposed as a key to promoting better mental health. In Chile, the Ministry of Education provided a socioemotional self-care support manual for teachers (called “teachers logbook”), which was voluntary and self-applied, and which included various exercises associated with seeking meaning in work, mindfulness and awareness of one’s own emotions, practicing self-compassion and emotional self-regulation, and taking care of personal relationships. Specifically, the four keys proposed by this manual to work on well-being are: (1) living according to a purpose, (2) recovering energy for well-being, (3) conducting one’s own behaviors and emotions, and (4) taking care of relationships [37]. However, its effect remains unevaluated. This self-care manual was available in an online format, although it was also distributed in a physical format.

Finally, it is necessary to mention that there is currently a knowledge gap on how spirituality, the use of socioemotional self-care strategies and life impact have affected teacher well-being and the possible mediating role of teachers’ mental health among them. Consequently, the present study tries to clarify this.

The return to face-to-face work alone does not erase the accumulated effects on teachers’ mental health. Therefore, it is necessary to know this relationship in order to adjust public policies to the consequences of the pandemic that will continue to manifest themselves for at least a couple of years after the pandemic is over.

## 2. Materials and Methods

### 2.1. Method

The Ethics Committee of Universidad de Tarapacá approved this research. An informed consent form was used, explaining the purpose, implications, scope and voluntary nature of participation. The methodology was non-experimental, since no variables were manipulated, and cross-sectional since the information was collected at a single point in time [38].

### 2.2. Participants

It was a non-random sample in which the entire teaching population of the city of Arica in Northern Chile was invited to respond to the instruments. The sample consisted of 624 teachers. A total of 74.4% were women and 25.6% were men. The mean age was 44.1 and the standard deviation was 11.9. A total of 56.4% belonged to public educational institutions and 43.6% belonged to subsidized institutions. A total of 99.4% were of Chilean nationality and the remaining 0.6% were of other nationalities. A total of 29% identified themselves as Aymara, 8% as Mapuche, 5.9% as Afro-descendant. A total of 56.4% did not identify with any ethnic group, while 87% said they were believers and 12% were non-believers.

### 2.3. Procedure

The total population of teachers in the region was invited to participate. All teachers were invited to participate in the study through the official channels of the regional Ministry of Education. The invitation was sent to all principals with a copy to their school coexistence teams. All teachers had the opportunity to participate in the online survey, clarifying that it was voluntary and anonymous. The approximate response time was 20 min.

### 2.4. Data Analysis

Initially, the demographic descriptions of the sample were analyzed, followed by the basic statistics of each variable, followed by the correlations between the variables. Structural equation analysis was then performed. SPSS version 22 and MPLUS version 8.4 were used. Missing values were replaced by multiple imputation (MI), considering seven imputed databases.

In the assessment of the structural equations, the classical criteria for the interpretation of model fit were taken as the reference, considering a Root Mean Square Error of Approximation (RMSEA) under 0.08 as acceptable and under 0.06 as good, while a Comparative Fit (CFI) and a Tucker-Lewis Index (TLI) greater than 0.9 was valued as adequate and over 0.95 as optimal [30,31,32]. Since chi-square is not currently considered an essential indicator given the problems it presents with samples larger than 200 and in cases of non-normal distribution, it was not considered. A SRMR (Standardized Root Mean-Square) under 0.8 was assessed as adequate. A χ^2^/df ratio was considered adequate under 3, although it is subject to the same limitation as Chi-square in large samples [39,40,41]. The Weighted Least Squares Robust Weighted Least Squares (WLSMV) estimator was used.

### 2.5. Instruments

Sociodemographic data were assessed through an ad hoc scale that asked about sex, age, nationality, ethnicity, religion and being a believer or not.

#### 2.5.1. General Life Satisfaction

Diener, Emmons, Larsen and Griffin [42] scale was used. This has 5 Likert scale items ranging from “strongly disagree” to “strongly agree”. An example item is “My life is similar to the life I would like to have”. A higher score indicates greater satisfaction with life. This scale was validated in an adult population in Chile, obtaining a unidimensional structure and a reliability α = 0.87 [43]. In the present study the Cronbach’s alpha was α = 0.85.

#### 2.5.2. Specific Life Satisfaction

It was measured through the adaptation of the Brief Multidimensional Student Satisfaction with Life Satisfaction Scale (BMSLSS) [44]. It has seven response options ranging from 1 = terrible to 7 = excellent. An example item is “I would describe my satisfaction with my family life as …”. The specific areas included are family life, friendships, work, neighborhood, and self. The only area that had to be replaced was school by work. Originally this scale was developed for students but given that these same dimensions are applicable to adults, replacing school with work, it was considered appropriate to use it as a complementary measure of life satisfaction. The validation in Chile of this instrument was conducted in schoolchildren and obtained a Cronbach reliability of 0.70 with a unidimensional structure [45]. In the present study, the Cronbach’s alpha was α = 0.82.

#### 2.5.3. Areas Affected by COVID-19

These were measured with five ad hoc likert scale questions between not at all and very much that included: worries, social relationships, economic situation, work and family life. Summation is considered as they are feasible to be assessed by any person. A higher score indicates that each area was more affected. The validation of the content of the items of this scale was carried out by two expert judges. The reliability of this scale was α = 0.78.

#### 2.5.4. COVID-19 Background

This information was evaluated through ad hoc questions with three options “No”, “not sure” and “yes”. These questions addressed the existence of personal history of infection, family history of infection, history of contact with infected persons, death of a family member or friend, and perceived risk of infection. Each question was considered separately in the analyses given the importance of assessing its content separately.

#### 2.5.5. Depression Anxiety Stress Scales (DASS-21)

This instrument measures depression, anxiety and stress, with a total of 21 Likert scale items [46]. It has four response options, from 0 (“does not describe anything that happened to me or that I felt during the week”) to 3 (“yes, this happened to me a lot most of the time”). A higher score indicates a greater presence of this symptomatology. It was validated in the adult population in Chile, obtaining a three-factor structure. The depression subscale obtained an Alpha of 0.85, while the stress subscale obtained an Alpha of 0.83 and the stress-anxiety subscale an Alpha of 0.73 [47]. Evidence of concurrent validity was also obtained. In the present study, these scales obtained the following reliability values for depression α = 0.89, anxiety α = 0.90, and stress α = 0.92.

#### 2.5.6. Use of Self-Applied Social-Emotional Strategies by Teachers

This question concerned the use of a self-application manual called *Teacher’s logbook*. Interviewees were asked whether they had used it at least once versus never having used it. Among those who had not used it, they were asked if they knew about it even if they had never used it.

#### 2.5.7. Brief Spirituality Scale

This was constructed by the lead author of the present research based on Koenig’s [48,49] reviews of essential aspects of spirituality, not necessarily including religiosity. It is a Likert scale instrument with five items ranging from strongly disagree to strongly agree. The questions it contains are “I believe in a higher intelligence or power.” “I feel that there is something more than what can be seen with the naked eye”. “I try to connect with a higher power or intelligence.” “I believe that a higher power or intelligence can help us in everyday life.” “Believing in a higher power helps me cope with problems.” The items were validated by three expert judges. A higher score indicates greater spirituality. The Cronbach’s alpha reliability was 0.94.

## 3. Results

Some 31.6% of the sample had used the teaching log at least once. Among those who had never used it, 15.1% said they had never heard of it. Meanwhile, 53.4% acknowledged having heard of it, but still not having used it. Table 1 shows the descriptive data of the variables. The skewness and kurtosis of all variables were found to be within the acceptable range to be considered normally distributed [50].

Table 2 presents the correlations between the study variables, most of which are significant. Both measures of life satisfaction correlate significantly with each other and with each of the DASS21 dimensions, as well as with spirituality, having a family history of infection and death of a family member or friend.

Contact with infected persons did not correlate significantly with satisfaction with life, although it did correlate with the DASS21 dimensions. Educational dependence also showed no significant relationship with any of the variables.

Using as a criterion that the variables have a significant relationship of at least 0.01 with any of the criterion variables or with the mediating variable, it can be observed that perceived risk is left out of the structural equation model in the following step, since it is only significant at 0.05.

### Structural Equations Model

**Figure 1:**
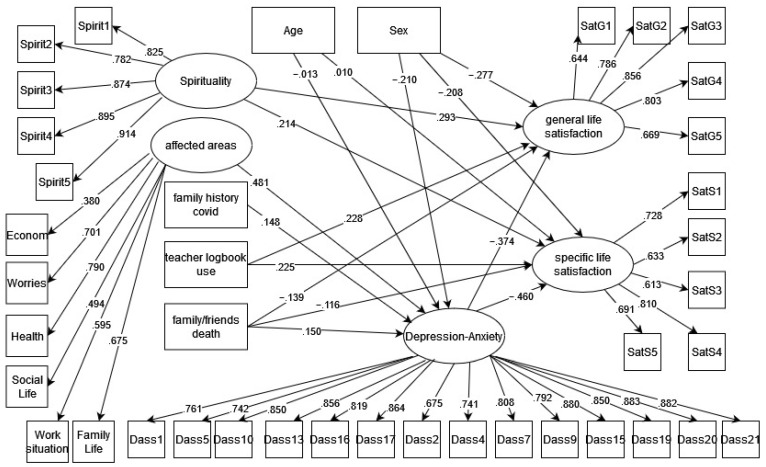
Legends: Structural equation model of the mental health mediation between spirituality/COVID-19 background/use of social-emotional self-care strategies and teacher well-being. The standardized values of the structural equation are presented. Each latent variable goes with its respective items.

Figure 1 presents the resulting structural equation model. The depression and anxiety subscales were considered as a single latent variable, leaving out all items of the stress subscale.

Age has a positive direct effect on specific life satisfaction of 0.010 (*p* < 0.01), as well as a negative direct effect on depression-anxiety of −0.013 (*p* < 0.001).

Sex has a negative direct effect on general life satisfaction of −0.277 (*p* < 0.001) and an indirect effect, through depression-anxiety, on general life satisfaction of 0.078 (*p* < 0.05). Additionally, it has a direct effect on specific life satisfaction of −0.208 (*p* < 0.05) and an indirect effect on specific life satisfaction of 0.096 (*p* < 0.05). Thus, being female is directly associated with greater life satisfaction. Sex has a negative direct effect on depression–anxiety of −0.210 (*p* < 0.05). Thus, being female is directly associated with greater depression and anxiety symptomatology.

Spirituality has a positive direct effect on general life satisfaction of 0.293 (*p* < 0.001) and on specific life satisfaction of 0.214 (*p* < 0.001), while no effect was identified on depression–anxiety.

Areas affected by COVID-19 have no direct effect on general or specific life satisfaction but have an indirect effect on general life satisfaction of −0.180 (*p* < 0.001) and on specific life satisfaction of −0.221 (*p* < 0.001). Affected areas have a positive direct effect on depression–anxiety of 0.481 (*p* < 0.001).

Family history of COVID-19 has a positive direct effect on depression–anxiety of 0.148 (*p* < 0.001), but no direct effect on general or specific life satisfaction.

Teacher logbook use has a positive direct effect on general life satisfaction of 0.228 (*p* < 0.001) and also on specific life satisfaction of 0.225 (*p* < 0.001). It has no effect on depression–anxiety.

Depression–anxiety has a negative direct effect on general life satisfaction of −0.374 (*p* < 0.001) and on specific life satisfaction of −0.460 (*p* < 0.001).

The death of a family member or friend has a negative direct effect on general life satisfaction of −0.139 (*p* < 0.01) and on specific life satisfaction of −0.116 (*p* < 0.01). Additionally, the death of a family member or friend has a positive direct effect on depression–anxiety of 0.150 (*p* < 0.01).

The goodness-of-fit indicators of the model were adequate: χ^2^/gl = 2.146, RMSEA = 0.043 [0.040–0.046], CFI = 0.951 and TLI = 0.947, SRMR = 0.075.

## 4. Discussions

The aim of this study was to analyze the vital impact of COVID-19, spirituality and the use of social-emotional self-care strategies on teacher well-being, mediated by mental health.

Although three dimensions of mental health were initially considered, the stress dimension was left out of the final model, with mental health being represented only by depression and anxiety items together. It is noteworthy that mental health is fully mediated between the areas affected by COVID-19 and life satisfaction, whether it is considered general or specific, and the same is true for family history of COVID-19. Consequently, the question arises as to why there is no direct effect of COVID-19-affected areas or family history of COVID-19 contagion on life satisfaction.

Contrary to expectations, spirituality does not have a direct effect on mental health, so there is no mediating effect of the latter. However, spirituality does have a direct effect on life satisfaction, both in general and specifically. This is only partially consistent with previous research [35,36]. Of course, it is possible that our spirituality instrument may not be sensitive enough in relation to mental health, but it is sensitive enough in relation to satisfaction with life

We also did not find a mental health mediating effect between teacher logbook use and satisfaction with life either general or specific. It is important to note that this is the first study to investigate the usefulness of this tool provided by the Chilean Ministry of Education. It is possible that some difficulties in its promotion or implementation in educational establishments have diminished its use among teachers. Having to use it in a space and time outside work hours may have discouraged its use. These are precisely some of the reasons why this tool has been criticized, as it remains at the level of individual responsibility for caring for the well-being of teachers, leaving out the broader responsibility of the entire school community [51]. However, in any case, its use seems to have a significant effect on teacher well-being.

A more complex view, which considers both individual teacher and school contextual aspects of their well-being at school, received more attention recently, considering that some sociodemographic aspects may also play an important role [52]. It is possible to consider that self-care is both individual and community. Similarly, it has been proposed that resilience is not only an individual construct, but also has a contextual level [53]. In the latter sense, the leadership of principals and managers may also be decisive in the adequate implementation of self-care of teachers [54].

Paying attention to the socioemotional base that teachers have is fundamental [55]. However, without socioemotional training of undergraduate teachers, one cannot seriously expect them to seek to value and take advantage of self-care resources that may be available in educational communities and/or government institutions. The alternative is to rely on the emotional intelligence that teachers spontaneously bring, which does not seem to be a desirable option considering all that is at stake in affecting so many aspects of the lives of students and teachers themselves [55].

On the other hand, teachers who experience burnout are often perceived as having lower socioemotional competence by their students. This suggests that students notice when their teachers are not doing well emotionally [56].

Regarding demographic variables, being a woman is associated with greater life satisfaction, contrary to what was found in Chile by Cabezas et al. [26], although the latter authors used a single question to measure well-being. On the other hand, in the present study, being a woman was associated with greater mental health problems. This coincides with the findings of Lizana and Lera [27]. However, unlike the latter study, no differences were found in the present study between public and subsidized schools. It is possible that this difference is partly explained by the fact that in Arica, most subsidized educational establishments are free for students, as are the public ones. This is different in Central Chile, where subsidized schools usually imply a higher cost for parents. Thus, the most vulnerable students remain in public or subsidized establishments indifferently, which could mean that the stress of attending more vulnerable students does not make a difference in this Northern Chilean city. In any case, it is striking that being a woman is associated with both greater satisfaction with life and greater symptomatology. It may seem contradictory, although it is possible that women may handle the symptomatology they experience differently. In this sense, it is possible that variables such as resilience and coping are key to understanding these results. However, this apparently coincides with the findings of Klapproth et al. [57], who observed that female teachers experience higher levels of stress but are better able to cope with it. A sense of mastery in having successfully coped with the pandemic may also contribute to teachers’ well-being [54].

Age was only positively associated with specific, but not overall, life satisfaction. It was also associated with poorer mental health. Thus, older age increases metal health problems. In this respect it is consistent with previous research on teachers [7]. It is possible that this result is affected by the COVID-19 contingency, since older age may be associated with the COVID-19 risk group and this is an additional stressor.

Mental health presented a partial mediation effect between the death of a family member or friend and both types of life satisfaction; thus, there is both a direct effect of the death of a family member or friend on both types of satisfaction, as well as an indirect effect through mental health. Interestingly, having a history of COVID-19 infection was not significantly associated with either mental health or either type of life satisfaction. It is possible that having contracted COVID-19 may be a relief for some, having survived the disease, while for others it may be generating side effects on physical health that have an effect on their mental health. This mixture could generate a null correlation between these variables. In contrast, family history of COVID-19 infection does have a significant effect on mental health, but not on life satisfaction. It is possible that having a family member infected with COVID-19 increases anxiety about contracting it, but also the concern about the possible death of this family member.

Although the emotional demands teachers receive from students existed prior to the pandemic, the need for self-care on the part of teachers-in-training has not been prioritized [58]. Despite the fact that as students in this career approach the end of it, their levels of well-being decrease [59]. This may indicate the need to sensitize teachers on this issue from their training, in addition to providing them with the strategies themselves. In the present study, it was evident that despite having a resource manual, a large number of teachers have not used it even once.

On the other hand, teachers with better emotional resources are useful models for students to develop their own emotional resources, especially if social-emotional learning is implemented in their schools. However, component 4 of the teacher logbook, taking care of relationships, becomes especially relevant if one considers the impact that having valuable relationships with students has on improving teachers’ well-being and decreasing their emotional exhaustion [60].

This study has some limitations. Each of the four components of the teaching logbook was not analyzed to discover which might have the greatest effect. Although there is no random selection, all teachers in Arica were invited to participate, so self-selection is an important element. Another limitation is that the use of the teacher logbook was considered to be at least once, so in the future it would be desirable to establish a more precise range of uses, since it is possible that greater frequency is associated with better results.

A strength of the present study is the use of a comprehensive measure of at least three dimensions of mental health. In addition, according to Agyapong et al. [16], it is one of the main instruments used to measure depression, anxiety and stress. In the study by Nabe-Nielsen et al. [10], they only considered three items to measure metal health, specifically asking about stress, burnout and working conditions. On the other hand, the study by Cabezas et al. [26] considered only one item on psychological distress as a measure of psychological well-being. Only Lizana and Lera [27] used the same validated and broader instrument than the present research.

Future research should address the longitudinal aspect of teacher mental health, which would make it possible to see whether the public policies implemented are having an impact. Undoubtedly, it is necessary to adjust these public policies in order to safeguard the mental health and well-being of teachers. The teacher logbook adds to the tools that can contribute to teacher wellbeing. Chile’s most recent public policy, called Let’s Be Community [61], pays attention to these aspects, but its effectiveness has not yet been evaluated.

## 5. Conclusions

Mental health has a mediating effect between the death of a close person, affected areas and family history with life satisfaction. Spirituality and the use of socio-emotional self-care strategies self-applied by the teachers had no direct relationship with their mental health, so their mediating effect in relation to life satisfaction was discarded. Teachers who used self-care social-emotional strategies, as well as those who reported higher levels of spirituality, obtained greater satisfaction with life, both in general and specifically. Women had higher levels of depression, anxiety and stress symptomatology, but also higher levels of life satisfaction.

## Figures and Tables

**Table 1:** Descriptive statistics.

	Minimum	Maximum	M	DS	Skewness	Kurtosis
Satisfaction with life General	5.0	35.0	26.8	5.5	−0.902	0.841
Specific life satisfaction	5.0	35.0	27.9	4.6	−0.963	1.606
Stress	7.0	28.0	15.1	5.9	0.311	−0.949
Anxiety	7.0	28.0	13.4	5.8	0.690	−0.611
Depression	7.0	28.0	13.1	5.1	0.810	0.032
Personal history of COVID-19 infection	1.0	3.0	1.8	0.4	−0.836	1.054
Family history of infection	1.0	3.0	1.7	0.9	0.597	−1.622
Contact with infected persons	1.0	3.0	1.5	0.8	1.078	−0.737
Perceived risk of infection	1.0	3.0	2.2	0.9	−0.429	−1.580
Death of a family member or friend	1.0	3.0	1.4	0.8	1.558	0.460
Areas affected by COVID-19	6.0	30.0	22.6	5.0	−0.663	0.149
Spirituality	5.0	30.0	22.3	7.9	−0.773	−0.638
Age	22.0	80.0	44.1	11.9	0.450	−0.644

**Table 2:** Correlations between study variables.

	1	2	3	4	5	6	7	8	9	10	11	12	13	14
1. General life satisfaction	-													
2. Specific life satisfaction	0.720 **	-												
3. Stress	−0.318 **	−0.427 **	-											
4. Anxiety	−0.296 **	−0.378 **	0.855 **	-										
5. Depression	−0.417 **	−0.502 **	0.807 **	0.791 **	-									
6. Personal history of COVID-19 infection	−0.051	−0.031	0.026	0.006	0.008	-								
7. Family history of infection	−0.129 **	−0.135 **	0.142 **	0.191 **	0.152 **	−0.175 **	-							
8. Contact with infected persons	−0.029	−0.037	0.088 *	0.081 *	0.090 *	−0.235 **	0.275 **	-						
9. Death of a relative or friend	−0.164 **	−0.146 **	0.144 **	0.156 **	0.131 **	−0.116 **	0.302 **	0.090 *	-					
10. Perceived risk of contagion	−0.081 *	−0.102 *	0.124 **	0.169 **	0.150 **	−0.058	0.159 **	0.133 **	0.136 **	-				
11. Spirituality	0.289 **	0.208 **	0.034	0.044	−0.024	−0.128 **	0.032	0.039	0.004	0.003	-			
12. Areas affected by COVID-19	−0.232 **	−0.280 **	0.418 **	0.402 **	0.394 **	−0.041	0.163 **	0.164 **	0.180 **	0.278 **	0.018	-		
13. Age	0.073	0.191 **	−0.224 **	−0.155 **	−0.151 **	−0.102 *	−0.041	−0.109 **	0.050	0.055	0.109 **	−0.136 **	-	
14. Sex	−0.083 *	−0.035	−0.135 **	−0.110 **	−0.083 *	0.014	−0.032	0.008	−0.018	0.037	−0.120 **	−0.088 *	0.002	-

* The correlation is significant at the 0.05 level (bilateral). ** Correlation is significant at the 0.01 level (bilateral).

## Data Availability

Data available on request due to restrictions. The data presented in this study are available on request from the corresponding author. The data are not publicly available due to they are the property of the Chilean government, having been obtained with public funds.
